# Tris(4-acetamido­phenoxy­methyl)methanol 0.7-hydrate

**DOI:** 10.1107/S1600536808032194

**Published:** 2008-10-25

**Authors:** Damon A. Parrish, Katie Juromski, Anne Marteel-Parrish, Reddy Damavarapu, Maoxi Zang, Dave Paritosh

**Affiliations:** a Laboratory for the Structure of Matter, Code 6030, Naval Research Laboratory, Washington, DC 20375, USA; b Washington College, 300 Washington Avenue, Chestertown, MD 21620, USA; c Geocenters, Inc., Building 3028, Picatinny Arsenal, NJ 07806-5000, USA

## Abstract

The asymmetric unit of the title compound, C_28_H_31_N_3_O_7_·0.7H_2_O, contains a mol­ecule of tris­(4-acetamido­phenoxy­meth­yl)methanol and 0.7 of a water mol­ecule. An extensive hydrogen-bonding network includes inter­actions between all components of the crystal structure.

## Related literature

For related structures, see: Haisa *et al.* (1980 ▶).
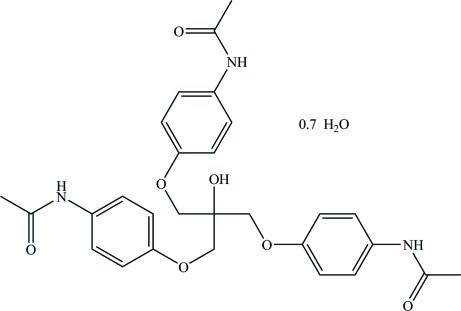


## Experimental

### 

#### Crystal data

C_28_H_31_N_3_O_7_·0.7H_2_O
*M* *_r_* = 534.17
Monoclinic, 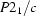

*a* = 9.4900 (9) Å
*b* = 29.992 (3) Å
*c* = 9.3879 (9) Å
β = 90.257 (2)°
*V* = 2672.0 (4) Å^3^
*Z* = 4
Mo *K*α radiation
μ = 0.10 mm^−1^
*T* = 103 (2) K
0.19 × 0.16 × 0.01 mm

#### Data collection

Bruker SMART APEXII CCD diffractometer
Absorption correction: multi-scan (*SADABS*; Bruker, 2004 ▶) *T*_min_ = 0.749, *T*_max_ = 1.000 (expected range = 0.748–0.999)
21154 measured reflections
4527 independent reflections
3113 reflections with *I* > 2σ(*I*)
*R*_int_ = 0.070

#### Refinement

*R*[*F* ^2^ > 2σ(*F* ^2^)] = 0.057
*wR*(*F* ^2^) = 0.131
*S* = 1.10
4527 reflections
363 parameters
2 restraints
H atoms treated by a mixture of independent and constrained refinement
Δρ_max_ = 0.24 e Å^−3^
Δρ_min_ = −0.25 e Å^−3^

### 

Data collection: *APEX2* (Bruker, 2006 ▶); cell refinement: *APEX2*; data reduction: *SAINT* (Bruker, 2002 ▶) and *XPREP* (Bruker, 2005 ▶); program(s) used to solve structure: *SHELXTL* (Sheldrick, 2008 ▶); program(s) used to refine structure: *SHELXTL*; molecular graphics: *SHELXTL*; software used to prepare material for publication: *SHELXTL*.

## Supplementary Material

Crystal structure: contains datablocks global, I. DOI: 10.1107/S1600536808032194/tk2305sup1.cif

Structure factors: contains datablocks I. DOI: 10.1107/S1600536808032194/tk2305Isup2.hkl

Additional supplementary materials:  crystallographic information; 3D view; checkCIF report

## Figures and Tables

**Table 1:** Hydrogen-bond geometry (Å, °)

*D*—H⋯*A*	*D*—H	H⋯*A*	*D*⋯*A*	*D*—H⋯*A*
O1—H1⋯O13*A*^i^	0.84	1.97	2.798 (3)	167
O1*S*—H1*SB*⋯O13*A*^ii^	0.85 (4)	2.00 (4)	2.842 (4)	168 (4)
N10*C*—H10*A*⋯O13*C*^iii^	0.88	1.95	2.812 (4)	167
N10*B*—H10*B*⋯O13*B*^iv^	0.88	1.95	2.824 (4)	175
N10*A*—H10*C*⋯O3*C*^v^	0.88	2.36	3.197 (3)	159
O1*S*—H1*SA*⋯O13*C*	0.85 (4)	1.98 (5)	2.817 (5)	167 (5)

Symmetry codes: (i) 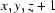; (ii) 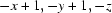; (iii) 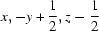; (iv) 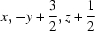; (v) 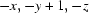.

**Fig. 1.**
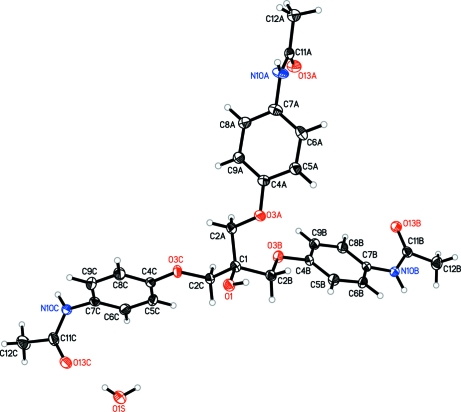
View of (I) showing the atom labeling for the non-hydrogen atoms. Displacement ellipsoids are shown at the 50% probability level.

## supplementary crystallographic information

### Comment

Tris(4-acetamidophenoxymethyl) methanol is one of the key ingredients of a cast-curable explosive formulation. The material exists as a liquid when hot and can be poured into a mold of any desired shape. Upon cooling, the material forms a highly stable solid explosive. The title compound has been characterized crystallographically as a 0.7 water solvate, (I), Fig. 1. The derived geometric parameters are comparable to those observed for the related compound N-(4-Methoxyphenyl)acetamide (Haisa *et al.*,  1980). The crystal structure of (I) is stabilized by an extensive network of hydrogen bonding interactions, Table 1.

### Experimental

4-Acetamidophenol (179 mg, 1.19 mmol) and 2,2-bis(chloromethyloxirane) (54 mg, 0.38 mmol) were heated at 90 °C in the presence of K_2_CO_3_ (197 mg, 1.43 mmol) in acetonitrile (5 ml) for 22 h. After cooling to room temperature, the reaction mixture was poured into water (5 ml) and the precipitate was extracted with ethyl acetate (3 × 20 ml, if the precipitate can not be dissolved in ethyl acetate completely, about 5 ml of acetone was added to improve the solubility). The organic phase was washed with water (10 ml) and dried over Na_2_SO_4_. The solvent was evaporated *in vacuo* and the residue was recrystallized from hot ethyl acetate. The molecule was obtained as a white solid, 82 mg (44%); m.p. = 486 K, ^1^H NMR (DMSO-d_6_) δ 9.75 (s, 3H), 7.45 (d, *J* = 8.8 Hz, 6H), 6.88 (d, *J* = 8.8 Hz, 6H), 5.50 (bs, 1H), 4.09 (s, 6H), 1.99 (s, 9H) p.p.m. ^13^C NMR (CDCl_3_) δ 168.1, 154.8, 133.2, 120.8, 115.1, 72.9, 69.7, 24.2 p.p.m. A clear colorless crystal of (I) was grown by slow evaporation from ethyl acetate and characterized as a 0.7 hydrate (from fractional refinement).

### Refinement

The non-water H atoms were included in the riding model approximation with O—H = 0.84, N—H = 0.88 and C—H = 0.95–0.99 Å, and with *U*(H) set to 1.2–1.5*U*_eq_(O, N and C). The population of the solvent water molecule was allowed to refine. The result was a population of 0.70. The H atoms were refined with O—H = 0.850 (1) and with *U*(H) = 1.2*U*_eq_(O).

### Crystal data

**Table:**

C_28_H_31_N_3_O_7_·0.7H_2_O	*Z* = 4
*M**_r_* = 534.17	*F*(000) = 1132
Monoclinic, *P*2_1_/*c*	*D*_x_ = 1.328 Mg m^−^^3^
Hall symbol: -P 2ybc	Mo *K*α radiation, λ = 0.71073 Å
*a* = 9.4900 (9) Å	µ = 0.10 mm^−^^1^
*b* = 29.992 (3) Å	*T* = 103 K
*c* = 9.3879 (9) Å	Plate, colourless
β = 90.257 (2)°	0.19 × 0.16 × 0.01 mm
*V* = 2672.0 (4) Å^3^	

### Data collection

**Table:**

Bruker SMART APEXII CCD diffractometer	4527 independent reflections
Radiation source: fine focus sealed tube	3113 reflections with *I* > 2σ(*I*)
graphite	*R*_int_ = 0.070
ω scans	θ_max_ = 24.7°, θ_min_ = 1.4°
Absorption correction: multi-scan (SADABS; Bruker, 2004)	*h* = −11→11
*T*_min_ = 0.749, *T*_max_ = 1.000	*k* = −35→35
21154 measured reflections	*l* = −11→9

### Refinement

**Table:**

Refinement on *F*^2^	Primary atom site location: structure-invariant direct methods
Least-squares matrix: full	Secondary atom site location: difference Fourier map
*R*[*F*^2^ > 2σ(*F*^2^)] = 0.057	Hydrogen site location: inferred from neighbouring sites
*wR*(*F*^2^) = 0.131	H atoms treated by a mixture of independent and constrained refinement
*S* = 1.10	*w* = 1/[σ^2^(*F*_o_^2^) + (0.0231*P*)^2^ + 4.8705*P*] where *P* = (*F*_o_^2^ + 2*F*_c_^2^)/3
4527 reflections	(Δ/σ)_max_ < 0.001
363 parameters	Δρ_max_ = 0.24 e Å^−^^3^
2 restraints	Δρ_min_ = −0.25 e Å^−^^3^

### Special details

**Table:**

Geometry. All e.s.d.'s (except the e.s.d. in the dihedral angle between two l.s. planes) are estimated using the full covariance matrix. The cell e.s.d.'s are taken into account individually in the estimation of e.s.d.'s in distances, angles and torsion angles; correlations between e.s.d.'s in cell parameters are only used when they are defined by crystal symmetry. An approximate (isotropic) treatment of cell e.s.d.'s is used for estimating e.s.d.'s involving l.s. planes.
Refinement. Refinement of *F*^2^ against ALL reflections. The weighted *R*-factor *wR* and goodness of fit *S* are based on *F*^2^, conventional *R*-factors *R* are based on *F*, with *F* set to zero for negative *F*^2^. The threshold expression of *F*^2^ > σ(*F*^2^) is used only for calculating *R*-factors(gt) *etc*. and is not relevant to the choice of reflections for refinement. *R*-factors based on *F*^2^ are statistically about twice as large as those based on *F*, and *R*- factors based on ALL data will be even larger.

### Fractional atomic coordinates and isotropic or equivalent isotropic displacement parameters (Å^2^)

**Table:**

	*x*	*y*	*z*	*U*_iso_*/*U*_eq_	Occ. (<1)
O1	0.2725 (2)	0.50948 (7)	0.3966 (2)	0.0218 (5)	
H1	0.2221	0.5325	0.4010	0.026*	
C1	0.3672 (3)	0.51325 (10)	0.2790 (4)	0.0185 (7)	
O1S	0.8382 (4)	0.34434 (13)	0.7394 (4)	0.0400 (14)	0.700 (7)
H1SA	0.774 (4)	0.3262 (15)	0.713 (6)	0.048*	0.700 (7)
H1SB	0.848 (6)	0.3678 (11)	0.690 (5)	0.048*	0.700 (7)
C2A	0.2906 (3)	0.50761 (10)	0.1358 (4)	0.0204 (7)	
H2AA	0.3598	0.5039	0.0581	0.025*	
H2AB	0.2294	0.4809	0.1383	0.025*	
C2B	0.4378 (3)	0.55887 (10)	0.2908 (4)	0.0199 (7)	
H2BA	0.3652	0.5825	0.2948	0.024*	
H2BB	0.4948	0.5604	0.3792	0.024*	
C2C	0.4724 (3)	0.47572 (10)	0.2976 (4)	0.0191 (7)	
H2CA	0.5415	0.4760	0.2188	0.023*	
H2CB	0.5241	0.4791	0.3888	0.023*	
O3C	0.3941 (2)	0.43482 (7)	0.2967 (2)	0.0213 (5)	
O3B	0.5258 (2)	0.56558 (7)	0.1702 (2)	0.0217 (5)	
O3A	0.2078 (2)	0.54665 (7)	0.1123 (2)	0.0228 (5)	
C4C	0.4656 (3)	0.39563 (10)	0.3235 (3)	0.0191 (7)	
C4B	0.5890 (3)	0.60715 (10)	0.1605 (4)	0.0183 (7)	
C4A	0.1464 (3)	0.55113 (11)	−0.0198 (4)	0.0194 (7)	
C5B	0.5688 (3)	0.64155 (10)	0.2563 (4)	0.0208 (8)	
H5BA	0.5047	0.6382	0.3330	0.025*	
C5C	0.6078 (3)	0.39279 (11)	0.3552 (4)	0.0216 (8)	
H5CA	0.6641	0.4189	0.3592	0.026*	
C5A	0.0895 (3)	0.59256 (11)	−0.0490 (4)	0.0237 (8)	
H5AA	0.0981	0.6160	0.0186	0.028*	
C6B	0.6432 (3)	0.68105 (10)	0.2392 (4)	0.0202 (7)	
H6BA	0.6286	0.7049	0.3041	0.024*	
C6C	0.6673 (4)	0.35123 (11)	0.3811 (4)	0.0228 (8)	
H6CA	0.7648	0.3491	0.4032	0.027*	
C6A	0.0198 (3)	0.59996 (11)	−0.1769 (4)	0.0242 (8)	
H6AA	−0.0184	0.6286	−0.1971	0.029*	
C7B	0.7386 (3)	0.68620 (10)	0.1289 (3)	0.0169 (7)	
C7C	0.5864 (3)	0.31298 (10)	0.3752 (3)	0.0193 (7)	
C7A	0.0054 (3)	0.56569 (11)	−0.2758 (4)	0.0218 (8)	
C8C	0.3838 (4)	0.35737 (11)	0.3158 (4)	0.0247 (8)	
H8CA	0.2865	0.3595	0.2926	0.030*	
C8B	0.7549 (4)	0.65171 (11)	0.0316 (4)	0.0233 (8)	
H8BA	0.8179	0.6550	−0.0460	0.028*	
C8A	0.0660 (4)	0.52477 (12)	−0.2465 (4)	0.0278 (8)	
H8AA	0.0586	0.5014	−0.3145	0.033*	
C9B	0.6799 (4)	0.61262 (11)	0.0474 (4)	0.0230 (8)	
H9BA	0.6908	0.5893	−0.0202	0.028*	
C9C	0.4435 (4)	0.31615 (11)	0.3418 (4)	0.0257 (8)	
H9CA	0.3873	0.2900	0.3368	0.031*	
C9A	0.1373 (4)	0.51726 (11)	−0.1193 (4)	0.0269 (8)	
H9AA	0.1796	0.4891	−0.1009	0.032*	
N10C	0.6469 (3)	0.26989 (9)	0.3956 (3)	0.0226 (7)	
H10A	0.6636	0.2533	0.3202	0.027*	
N10B	0.8192 (3)	0.72619 (8)	0.1210 (3)	0.0198 (6)	
H10B	0.8368	0.7399	0.2021	0.024*	
N10A	−0.0753 (3)	0.57205 (10)	−0.4032 (3)	0.0264 (7)	
H10C	−0.1676	0.5699	−0.3983	0.032*	
C11C	0.6794 (4)	0.25360 (11)	0.5244 (4)	0.0266 (8)	
C11B	0.8710 (3)	0.74511 (11)	0.0031 (4)	0.0205 (8)	
C11A	−0.0173 (4)	0.58104 (10)	−0.5292 (4)	0.0201 (8)	
C12A	−0.1164 (4)	0.58705 (12)	−0.6517 (4)	0.0314 (9)	
H12A	−0.0723	0.5760	−0.7390	0.047*	
H12B	−0.1386	0.6188	−0.6628	0.047*	
H12C	−0.2033	0.5703	−0.6338	0.047*	
C12B	0.9471 (4)	0.78871 (11)	0.0263 (4)	0.0317 (9)	
H12D	1.0185	0.7926	−0.0478	0.048*	
H12E	0.9928	0.7885	0.1201	0.048*	
H12F	0.8793	0.8133	0.0216	0.048*	
C12C	0.7326 (5)	0.20622 (12)	0.5285 (4)	0.0425 (11)	
H12G	0.8116	0.2041	0.5957	0.064*	
H12H	0.7643	0.1975	0.4333	0.064*	
H12I	0.6566	0.1863	0.5591	0.064*	
O13B	0.8573 (3)	0.72890 (8)	−0.1172 (3)	0.0296 (6)	
O13A	0.1115 (2)	0.58529 (8)	−0.5433 (3)	0.0280 (6)	
O13C	0.6634 (3)	0.27573 (8)	0.6346 (3)	0.0339 (6)	

### Atomic displacement parameters (Å^2^)

**Table:**

	*U*^11^	*U*^22^	*U*^33^	*U*^12^	*U*^13^	*U*^23^
O1	0.0250 (13)	0.0198 (12)	0.0207 (13)	0.0008 (10)	0.0025 (10)	0.0016 (10)
C1	0.0193 (17)	0.0174 (17)	0.0186 (19)	0.0005 (14)	0.0012 (14)	0.0024 (14)
O1S	0.059 (3)	0.034 (3)	0.027 (3)	−0.013 (2)	0.001 (2)	0.0084 (18)
C2A	0.0218 (18)	0.0155 (17)	0.024 (2)	0.0035 (14)	−0.0047 (15)	0.0014 (14)
C2B	0.0248 (18)	0.0182 (17)	0.0167 (19)	0.0016 (14)	0.0021 (15)	0.0017 (14)
C2C	0.0220 (17)	0.0153 (16)	0.0200 (19)	−0.0026 (14)	−0.0029 (14)	0.0026 (14)
O3C	0.0207 (12)	0.0131 (11)	0.0301 (14)	−0.0012 (10)	−0.0063 (10)	0.0029 (10)
O3B	0.0276 (12)	0.0179 (12)	0.0196 (14)	−0.0022 (10)	0.0062 (10)	−0.0010 (10)
O3A	0.0289 (13)	0.0188 (12)	0.0207 (14)	0.0067 (10)	−0.0066 (10)	−0.0004 (10)
C4C	0.0268 (18)	0.0152 (17)	0.0153 (19)	0.0027 (14)	−0.0017 (14)	0.0004 (14)
C4B	0.0222 (17)	0.0133 (16)	0.0193 (19)	−0.0002 (14)	−0.0020 (15)	0.0024 (14)
C4A	0.0184 (17)	0.0223 (18)	0.0176 (19)	0.0001 (14)	0.0001 (14)	0.0003 (15)
C5B	0.0214 (17)	0.0208 (17)	0.0200 (19)	−0.0015 (14)	0.0044 (15)	0.0006 (15)
C5C	0.0229 (18)	0.0163 (17)	0.026 (2)	−0.0040 (14)	−0.0040 (15)	−0.0008 (15)
C5A	0.0282 (19)	0.0209 (18)	0.022 (2)	0.0030 (15)	−0.0009 (16)	0.0004 (15)
C6B	0.0237 (18)	0.0149 (17)	0.022 (2)	0.0031 (14)	−0.0010 (15)	−0.0042 (14)
C6C	0.0235 (18)	0.0215 (19)	0.023 (2)	0.0034 (15)	−0.0040 (15)	−0.0019 (15)
C6A	0.0239 (18)	0.0216 (18)	0.027 (2)	0.0053 (15)	0.0028 (16)	0.0072 (16)
C7B	0.0203 (17)	0.0146 (16)	0.0158 (18)	0.0003 (13)	−0.0005 (14)	0.0021 (14)
C7C	0.0284 (19)	0.0171 (17)	0.0124 (18)	0.0040 (15)	0.0003 (14)	−0.0003 (14)
C7A	0.0140 (16)	0.031 (2)	0.020 (2)	−0.0007 (15)	0.0003 (14)	0.0051 (16)
C8C	0.0218 (18)	0.0222 (18)	0.030 (2)	−0.0009 (15)	−0.0044 (15)	0.0029 (16)
C8B	0.0311 (19)	0.0214 (18)	0.017 (2)	−0.0016 (16)	0.0071 (15)	0.0024 (15)
C8A	0.035 (2)	0.0242 (19)	0.025 (2)	0.0022 (16)	−0.0085 (17)	−0.0027 (16)
C9B	0.0315 (19)	0.0188 (18)	0.019 (2)	0.0031 (15)	0.0024 (16)	−0.0026 (14)
C9C	0.031 (2)	0.0189 (18)	0.027 (2)	−0.0048 (15)	−0.0006 (16)	−0.0007 (16)
C9A	0.034 (2)	0.0193 (18)	0.028 (2)	0.0061 (16)	−0.0109 (17)	−0.0016 (16)
N10C	0.0371 (17)	0.0143 (14)	0.0165 (16)	0.0061 (13)	−0.0005 (13)	−0.0032 (12)
N10B	0.0256 (15)	0.0167 (14)	0.0170 (16)	−0.0010 (12)	0.0000 (12)	−0.0006 (12)
N10A	0.0159 (14)	0.0404 (18)	0.0228 (18)	0.0002 (13)	−0.0046 (13)	0.0068 (14)
C11C	0.033 (2)	0.0179 (18)	0.029 (2)	0.0020 (16)	0.0021 (17)	0.0042 (16)
C11B	0.0226 (18)	0.0190 (17)	0.020 (2)	0.0037 (14)	0.0012 (15)	0.0016 (15)
C11A	0.0230 (19)	0.0137 (16)	0.023 (2)	0.0040 (14)	−0.0035 (15)	−0.0019 (14)
C12A	0.040 (2)	0.0263 (19)	0.028 (2)	0.0026 (17)	−0.0091 (17)	−0.0021 (17)
C12B	0.041 (2)	0.0231 (19)	0.031 (2)	−0.0065 (17)	0.0056 (18)	0.0015 (17)
C12C	0.070 (3)	0.024 (2)	0.033 (2)	0.015 (2)	−0.002 (2)	0.0031 (18)
O13B	0.0481 (16)	0.0227 (13)	0.0180 (14)	−0.0045 (12)	0.0022 (12)	0.0025 (11)
O13A	0.0245 (14)	0.0340 (14)	0.0254 (15)	−0.0010 (11)	−0.0015 (11)	0.0024 (11)
O13C	0.0609 (18)	0.0208 (13)	0.0200 (15)	0.0049 (12)	0.0006 (13)	0.0017 (11)

### Geometric parameters (Å, °)

**Table:**

O1—C1	1.431 (4)	C7B—C8B	1.389 (4)
O1—H1	0.8400	C7B—N10B	1.425 (4)
C1—C2C	1.515 (4)	C7C—C9C	1.394 (5)
C1—C2B	1.527 (4)	C7C—N10C	1.426 (4)
C1—C2A	1.534 (5)	C7A—C8A	1.382 (5)
O1S—H1SA	0.85 (4)	C7A—N10A	1.430 (4)
O1S—H1SB	0.85 (4)	C8C—C9C	1.381 (5)
C2A—O3A	1.427 (4)	C8C—H8CA	0.9500
C2A—H2AA	0.9900	C8B—C9B	1.380 (5)
C2A—H2AB	0.9900	C8B—H8BA	0.9500
C2B—O3B	1.425 (4)	C8A—C9A	1.389 (5)
C2B—H2BA	0.9900	C8A—H8AA	0.9500
C2B—H2BB	0.9900	C9B—H9BA	0.9500
C2C—O3C	1.434 (4)	C9C—H9CA	0.9500
C2C—H2CA	0.9900	C9A—H9AA	0.9500
C2C—H2CB	0.9900	N10C—C11C	1.338 (4)
O3C—C4C	1.380 (4)	N10C—H10A	0.8800
O3B—C4B	1.387 (4)	N10B—C11B	1.340 (4)
O3A—C4A	1.374 (4)	N10B—H10B	0.8800
C4C—C5C	1.383 (5)	N10A—C11A	1.334 (4)
C4C—C8C	1.387 (5)	N10A—H10C	0.8800
C4B—C9B	1.381 (5)	C11C—O13C	1.239 (4)
C4B—C5B	1.383 (4)	C11C—C12C	1.509 (5)
C4A—C5A	1.382 (4)	C11B—O13B	1.236 (4)
C4A—C9A	1.383 (5)	C11B—C12B	1.509 (5)
C5B—C6B	1.389 (4)	C11A—O13A	1.237 (4)
C5B—H5BA	0.9500	C11A—C12A	1.493 (5)
C5C—C6C	1.389 (5)	C12A—H12A	0.9800
C5C—H5CA	0.9500	C12A—H12B	0.9800
C5A—C6A	1.386 (5)	C12A—H12C	0.9800
C5A—H5AA	0.9500	C12B—H12D	0.9800
C6B—C7B	1.387 (5)	C12B—H12E	0.9800
C6B—H6BA	0.9500	C12B—H12F	0.9800
C6C—C7C	1.381 (5)	C12C—H12G	0.9800
C6C—H6CA	0.9500	C12C—H12H	0.9800
C6A—C7A	1.392 (5)	C12C—H12I	0.9800
C6A—H6AA	0.9500		
			
C1—O1—H1	109.5	C6C—C7C—N10C	121.6 (3)
O1—C1—C2C	105.6 (3)	C9C—C7C—N10C	118.8 (3)
O1—C1—C2B	107.0 (3)	C8A—C7A—C6A	118.9 (3)
C2C—C1—C2B	111.6 (3)	C8A—C7A—N10A	120.4 (3)
O1—C1—C2A	111.8 (3)	C6A—C7A—N10A	120.7 (3)
C2C—C1—C2A	109.2 (3)	C9C—C8C—C4C	120.1 (3)
C2B—C1—C2A	111.6 (3)	C9C—C8C—H8CA	119.9
H1SA—O1S—H1SB	116 (6)	C4C—C8C—H8CA	119.9
O3A—C2A—C1	107.7 (3)	C9B—C8B—C7B	120.2 (3)
O3A—C2A—H2AA	110.2	C9B—C8B—H8BA	119.9
C1—C2A—H2AA	110.2	C7B—C8B—H8BA	119.9
O3A—C2A—H2AB	110.2	C7A—C8A—C9A	121.1 (3)
C1—C2A—H2AB	110.2	C7A—C8A—H8AA	119.5
H2AA—C2A—H2AB	108.5	C9A—C8A—H8AA	119.5
O3B—C2B—C1	109.1 (3)	C8B—C9B—C4B	120.5 (3)
O3B—C2B—H2BA	109.9	C8B—C9B—H9BA	119.7
C1—C2B—H2BA	109.9	C4B—C9B—H9BA	119.7
O3B—C2B—H2BB	109.9	C8C—C9C—C7C	119.9 (3)
C1—C2B—H2BB	109.9	C8C—C9C—H9CA	120.0
H2BA—C2B—H2BB	108.3	C7C—C9C—H9CA	120.0
O3C—C2C—C1	107.1 (2)	C4A—C9A—C8A	119.4 (3)
O3C—C2C—H2CA	110.3	C4A—C9A—H9AA	120.3
C1—C2C—H2CA	110.3	C8A—C9A—H9AA	120.3
O3C—C2C—H2CB	110.3	C11C—N10C—C7C	122.9 (3)
C1—C2C—H2CB	110.3	C11C—N10C—H10A	118.6
H2CA—C2C—H2CB	108.6	C7C—N10C—H10A	118.6
C4C—O3C—C2C	118.2 (2)	C11B—N10B—C7B	126.8 (3)
C4B—O3B—C2B	115.8 (2)	C11B—N10B—H10B	116.6
C4A—O3A—C2A	116.8 (2)	C7B—N10B—H10B	116.6
O3C—C4C—C5C	124.8 (3)	C11A—N10A—C7A	123.2 (3)
O3C—C4C—C8C	114.8 (3)	C11A—N10A—H10C	118.4
C5C—C4C—C8C	120.4 (3)	C7A—N10A—H10C	118.4
C9B—C4B—C5B	120.0 (3)	O13C—C11C—N10C	122.0 (3)
C9B—C4B—O3B	115.4 (3)	O13C—C11C—C12C	121.7 (3)
C5B—C4B—O3B	124.5 (3)	N10C—C11C—C12C	116.3 (3)
O3A—C4A—C5A	115.5 (3)	O13B—C11B—N10B	123.4 (3)
O3A—C4A—C9A	124.3 (3)	O13B—C11B—C12B	121.4 (3)
C5A—C4A—C9A	120.2 (3)	N10B—C11B—C12B	115.1 (3)
C4B—C5B—C6B	119.3 (3)	O13A—C11A—N10A	121.9 (3)
C4B—C5B—H5BA	120.3	O13A—C11A—C12A	121.6 (3)
C6B—C5B—H5BA	120.3	N10A—C11A—C12A	116.5 (3)
C4C—C5C—C6C	119.2 (3)	C11A—C12A—H12A	109.5
C4C—C5C—H5CA	120.4	C11A—C12A—H12B	109.5
C6C—C5C—H5CA	120.4	H12A—C12A—H12B	109.5
C4A—C5A—C6A	120.0 (3)	C11A—C12A—H12C	109.5
C4A—C5A—H5AA	120.0	H12A—C12A—H12C	109.5
C6A—C5A—H5AA	120.0	H12B—C12A—H12C	109.5
C7B—C6B—C5B	121.0 (3)	C11B—C12B—H12D	109.5
C7B—C6B—H6BA	119.5	C11B—C12B—H12E	109.5
C5B—C6B—H6BA	119.5	H12D—C12B—H12E	109.5
C7C—C6C—C5C	120.9 (3)	C11B—C12B—H12F	109.5
C7C—C6C—H6CA	119.6	H12D—C12B—H12F	109.5
C5C—C6C—H6CA	119.6	H12E—C12B—H12F	109.5
C5A—C6A—C7A	120.3 (3)	C11C—C12C—H12G	109.5
C5A—C6A—H6AA	119.8	C11C—C12C—H12H	109.5
C7A—C6A—H6AA	119.8	H12G—C12C—H12H	109.5
C6B—C7B—C8B	118.9 (3)	C11C—C12C—H12I	109.5
C6B—C7B—N10B	119.0 (3)	H12G—C12C—H12I	109.5
C8B—C7B—N10B	122.1 (3)	H12H—C12C—H12I	109.5
C6C—C7C—C9C	119.5 (3)		
			
O1—C1—C2A—O3A	71.9 (3)	C5C—C6C—C7C—N10C	177.3 (3)
C2C—C1—C2A—O3A	−171.7 (2)	C5A—C6A—C7A—C8A	−2.3 (5)
C2B—C1—C2A—O3A	−47.8 (3)	C5A—C6A—C7A—N10A	175.3 (3)
O1—C1—C2B—O3B	−175.9 (2)	O3C—C4C—C8C—C9C	−179.5 (3)
C2C—C1—C2B—O3B	69.1 (3)	C5C—C4C—C8C—C9C	0.9 (5)
C2A—C1—C2B—O3B	−53.4 (3)	C6B—C7B—C8B—C9B	−1.7 (5)
O1—C1—C2C—O3C	59.4 (3)	N10B—C7B—C8B—C9B	176.7 (3)
C2B—C1—C2C—O3C	175.2 (3)	C6A—C7A—C8A—C9A	1.5 (5)
C2A—C1—C2C—O3C	−60.9 (3)	N10A—C7A—C8A—C9A	−176.1 (3)
C1—C2C—O3C—C4C	−174.9 (3)	C7B—C8B—C9B—C4B	−0.7 (5)
C1—C2B—O3B—C4B	175.3 (3)	C5B—C4B—C9B—C8B	2.3 (5)
C1—C2A—O3A—C4A	171.0 (3)	O3B—C4B—C9B—C8B	−176.3 (3)
C2C—O3C—C4C—C5C	1.8 (5)	C4C—C8C—C9C—C7C	−0.3 (5)
C2C—O3C—C4C—C8C	−177.8 (3)	C6C—C7C—C9C—C8C	−0.4 (5)
C2B—O3B—C4B—C9B	176.8 (3)	N10C—C7C—C9C—C8C	−177.4 (3)
C2B—O3B—C4B—C5B	−1.7 (4)	O3A—C4A—C9A—C8A	176.4 (3)
C2A—O3A—C4A—C5A	−168.4 (3)	C5A—C4A—C9A—C8A	−2.4 (5)
C2A—O3A—C4A—C9A	12.8 (5)	C7A—C8A—C9A—C4A	0.8 (5)
C9B—C4B—C5B—C6B	−1.5 (5)	C6C—C7C—N10C—C11C	78.5 (4)
O3B—C4B—C5B—C6B	176.9 (3)	C9C—C7C—N10C—C11C	−104.7 (4)
O3C—C4C—C5C—C6C	179.5 (3)	C6B—C7B—N10B—C11B	−151.8 (3)
C8C—C4C—C5C—C6C	−0.9 (5)	C8B—C7B—N10B—C11B	29.9 (5)
O3A—C4A—C5A—C6A	−177.2 (3)	C8A—C7A—N10A—C11A	−82.6 (4)
C9A—C4A—C5A—C6A	1.7 (5)	C6A—C7A—N10A—C11A	99.9 (4)
C4B—C5B—C6B—C7B	−0.9 (5)	C7C—N10C—C11C—O13C	−2.6 (5)
C4C—C5C—C6C—C7C	0.2 (5)	C7C—N10C—C11C—C12C	175.6 (3)
C4A—C5A—C6A—C7A	0.7 (5)	C7B—N10B—C11B—O13B	−1.9 (5)
C5B—C6B—C7B—C8B	2.5 (5)	C7B—N10B—C11B—C12B	177.3 (3)
C5B—C6B—C7B—N10B	−176.0 (3)	C7A—N10A—C11A—O13A	−1.3 (5)
C5C—C6C—C7C—C9C	0.5 (5)	C7A—N10A—C11A—C12A	−179.6 (3)

### Hydrogen-bond geometry (Å, °)

**Table:**

*D*—H···*A*	*D*—H	H···*A*	*D*···*A*	*D*—H···*A*
O1—H1···O13A^i^	0.84	1.97	2.798 (3)	167
O1S—H1SB···O13A^ii^	0.85 (4)	2.00 (4)	2.842 (4)	168 (4)
N10C—H10A···O13C^iii^	0.88	1.95	2.812 (4)	167
N10B—H10B···O13B^iv^	0.88	1.95	2.824 (4)	175
N10A—H10C···O3C^v^	0.88	2.36	3.197 (3)	159
O1S—H1SA···O13C	0.85 (4)	1.98 (5)	2.817 (5)	167 (5)

Symmetry codes: (i) *x*, *y*, *z*+1; (ii) −*x*+1, −*y*+1, −*z*; (iii) *x*, −*y*+1/2, *z*−1/2; (iv) *x*, −*y*+3/2, *z*+1/2; (v) −*x*, −*y*+1, −*z*.
